# Characterization of cytoplasmic viscosity of hundreds of single tumour cells based on micropipette aspiration

**DOI:** 10.1098/rsos.181707

**Published:** 2019-03-20

**Authors:** K. Wang, X. H. Sun, Y. Zhang, T. Zhang, Y. Zheng, Y. C. Wei, P. Zhao, D. Y. Chen, H. A. Wu, W. H. Wang, R. Long, J. B. Wang, J. Chen

**Affiliations:** 1 State Key Laboratory of Transducer Technology, Institute of Electronics, Chinese Academy of Sciences, Beijing, People's Republic of China; 2 School of Electronic, Electrical and Communication Engineering, University of Chinese Academy of Sciences, Beijing, People's Republic of China; 3 Department of Mechanical Engineering, University of Colorado, Boulder, CO, USA; 4 CAS Key Laboratory of Mechanical Behavior and Design of Materials, Department of Modern Mechanics, University of Science and Technology of China, Hefei, Anhui Province, People's Republic of China; 5 The Affiliated High School of Peking University, Beijing, People's Republic of China; 6 Department of Precision Instrument, Tsinghua University, Beijing, People's Republic of China

**Keywords:** cellular biophysics, micropipette aspiration, cytoplasmic viscosity, single tumour cells

## Abstract

Cytoplasmic viscosity (*μ*_c_) is a key biomechanical parameter for evaluating the status of cellular cytoskeletons. Previous studies focused on white blood cells, but the data of cytoplasmic viscosity for tumour cells were missing. Tumour cells (H1299, A549 and drug-treated H1299 with compromised cytoskeletons) were aspirated continuously through a micropipette at a pressure of −10 or −5 kPa where aspiration lengths as a function of time were obtained and translated to cytoplasmic viscosity based on a theoretical Newtonian fluid model. Quartile coefficients of dispersion were quantified to evaluate the distributions of cytoplasmic viscosity within the same cell type while neural network-based pattern recognitions were used to classify different cell types based on cytoplasmic viscosity. The single-cell cytoplasmic viscosity with three quartiles and the quartile coefficient of dispersion were quantified as 16.7 Pa s, 42.1 Pa s, 110.3 Pa s and 74% for H1299 cells at −10 kPa (*n*_cell_ = 652); 144.8 Pa s, 489.8 Pa s, 1390.7 Pa s, and 81% for A549 cells at −10 kPa (*n*_cell_ = 785); 7.1 Pa s, 13.7 Pa s, 31.5 Pa s, and 63% for CD-treated H1299 cells at −10 kPa (*n*_cell_ = 651); and 16.9 Pa s, 48.2 Pa s, 150.2 Pa s, and 80% for H1299 cells at −5 kPa (*n*_cell_ = 600), respectively. Neural network-based pattern recognition produced successful classification rates of 76.7% for H1299 versus A549, 67.0% for H1299 versus drug-treated H1299 and 50.3% for H1299 at −5 and −10 kPa. Variations of cytoplasmic viscosity were observed within the same cell type and among different cell types, suggesting the potential role of cytoplasmic viscosity in cell status evaluation and cell type classification.

## 1. Introduction

The mechanical behaviour of biological cells is largely determined by their cytoskeletons [1,2]. As tumour progresses, abnormal cellular functions of cancer cells can alter cytoskeletons, leading to increases in cell deformability and invading capabilities [3–5].

Well-established techniques that are being used to measure the mechanical properties of single tumour cells mainly include atomic force microscopy (AFM) and micropipette aspiration [4,6–8]. In AFM, a pyramidal or spherical probe tip attached to a flexible cantilever is pressed onto the cellular surface for a set distance and then the deflection of the cantilever is measured using a laser beam, which is used to estimate the stiffness of the probed surface based on contact mechanics models [9–19]. Although powerful, AFM has limited throughput and thus compromised performances for the acquisition of large data volume [20] (e.g. less than 10 cells per sample from patient pleural fluids [21]).

On the other hand, in micropipette aspiration, a portion of a single cell is aspirated into a small glass tube with the leading edge of its surface tracked and translated to cellular elastic and viscous properties [22,23]. Compared with AFM, micropipette aspiration involves deformations of larger cellular portions, and it can characterize the mechanical properties of single cells in a more global manner [24–28]. However, in conventional micropipette aspiration, a low pressure was used to aspirate a single tumour cell partially into the pipette and after that, the cell being measured would be expelled out of the pipette. This procedure takes a considerable amount of time and thus suffers from the limitation of low throughput.

In order to improve the detection throughput, a few previous studies achieved continuous micropipette aspiration by using high aspiration pressure and pipette diameters that are marginally smaller than the cell size [29–31]. Based on image processing, the aspiration lengths as a function of time were obtained and translated to cytoplasmic viscosity using a theoretical Newtonian fluid drop model. However, these previous studies only reported cytoplasmic viscosity of blood cells while the data of cytoplasmic viscosity of tumour cells were missing.

In this study, the same approach was adopted to characterize single tumour cells continuously and the corresponding values of cytoplasmic viscosity from hundreds of single tumour cells were reported for the first time. These values of cytoplasmic viscosity, which formed a statistically important dataset, were then used to classify two different cell types (A549 versus H1299) and the same cell type but with different status (H1299 cells with and without compromised cytoskeletons) in order to demonstrate the potentials of using cytoplasmic viscosity in cell type classification and cell status evaluation.

## 2. Material and methods

### 2.1. Working flow chart

**Figure 1.**
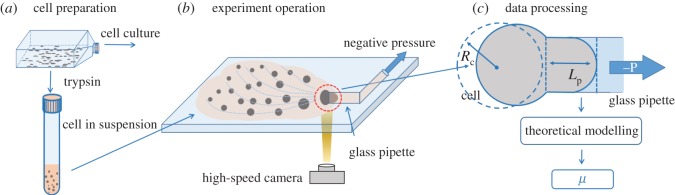
The working flow chart for continuously characterizing single-cell cytoplasmic viscosity (*μ*_c_) based on micropipette aspiration, which includes key steps of cell preparation, experimental operation and data processing. More specifically, during operations, cells in suspension were aspirated into a glass pipette continuously with cellular entry processes monitored by a high-speed camera. Based on image processing, raw data of cellular radius (*R*_c_), and aspiration length (*L*_p_) as a function of time were obtained. Leveraging a theoretical Newtonian liquid drop model, these raw data were translated to *μ*_c_ of single cells.

The working flow chart for continuously characterizing single-cell cytoplasmic viscosity based on micropipette aspiration includes three main steps: cell preparation, experimental operation and data processing (figure 1). During operations, cells in suspension were aspirated into a glass pipette continuously with cellular entry processes monitored by a high-speed camera. Based on image processing, raw data of cellular radius (*R*_c_), and aspiration length (*L*_p_) as a function of time were obtained. By combining experimental results with a theoretical Newtonian fluid drop model, cytoplasmic viscosity (*μ*_c_) of single cells was determined.

### 2.2. Materials, cell culture and drug treatment

Unless otherwise indicated, all cell-culture reagents were purchased from Life Technologies Corporation (Van Allen Way, Carlsbad, CA, USA). Lung tumour cell lines, A549 and H1299, were purchased from ATCC (The Global Bioresource Center, USA) and cultured with RPMI-1640 media supplemented with 10% fetal bovine serum and 1% penicillin and streptomycin. Immediately prior to an experiment, cells were trypsinized, centrifuged and resuspended in the supplemented culture medium with a concentration of 1 million cells ml^−1^.

Cytochalasin D (CD), a cell permeable fungal toxin that can depolymerize action filaments, was used in this study to compromise cytoskeletons following previously reported procedures [32]. More specifically, CD with a concentration of 1 µg ml^−1^ was added into the supplemented culture medium of H1299 for 30 min and then H1299 cells with compromised cytoskeletons (CD-treated H1299 cells) were trypsinized and characterized by micropipette for the quantification of cytoplasmic viscosity.

### 2.3. Experimental operation and raw data extraction

The experiments of micropipette aspiration were summarized as follows. A glass pipette with a diameter of 10 µm was fabricated by a micropipette puller (P-1000, Sutter Instruments, USA). During operation, single cells in suspension at a concentration of 10^6^ cells ml^−1^ were applied on a glass slide and the glass pipette was preloaded with culture medium. A negative pressure of 10 or 5 kPa was generated from a pressure controller (DPI-610, Druck, USA) to aspirate cells into the glass pipette, and the aspiration process was monitored by a high-speed camera (M320S, Phantom, USA) at a speed of 400–800 frames s^−1^.

In order to quantify cellular radius (*R*_c_), and aspiration length (*L*_p_) as a function of time, a sequence of image processing steps were used to track the process of cell elongation inside the glass pipette [33]. Briefly, the video of cell entry was firstly divided into multiple images at different time frames, and then key steps of frame differencing, thresholding, particle removal using erosion, edge detection and contour fitting were conducted sequentially.

### 2.4. Quantification of cytoplasmic viscosity

In this study, similar to most previous studies on micropipette aspiration [34], the theoretical Newtonian fluid model was used to model a single cell where the one-dimensional cellular aspiration into a micropipette was represented by the following equation [34]

2.1 $$\frac{R_{p}(\Delta P-P_{\mathrm{cr}})}{\mu_{c}(dL_{p}(t)/dt)}=6\left(1-\frac{R_{p}}{R_{c}}\right),$$

where Δ*P*, *P*_cr_, *R*_p_ are the aspiration pressure, critical pressure and pipette radius, respectively. In this study, Δ*P* and *R*_p_ were operational parameters in experiments and *L*_p_(*t*) and *R*_c_ were derived from image processing. As to *P*_cr_*,* it can be determined by the following equation:

2.2 $$P_{\mathrm{cr}}=2T_{0}\left(\frac{1}{R_{p}}-\frac{1}{R_{c}}\right),$$

where *T*_0_ is the cortical tension of the cell. In our experiments, *R*_p_ is about 5.0 µm and *R*_c_ is in the range of 6.0–12.5 µm. The cortical tension of tumour cells *T*_0_ was found to be of the order of 10^−5^ N m^−1^ in the literature [34]. Therefore, the critical pressure *P*_cr_ was estimated to be of the order of approximately 10 Pa. Unlike previous experiments where low aspiration pressure of the order of 10 Pa was used, here a high aspiration pressure of 5 or 10 kPa was used, which was 3 orders of magnitude higher than *P*_cr_. Therefore, *P*_cr_ was neglected in equation (2.1), which is consistent with previous publications where high aspiration pressures were used to characterize cytoplasmic viscosity of white blood cells [29–31]. It should be noted that the model above approximates the cell as a homogenized droplet enclosed by the cell membrane, and thereby the cytoplasmic viscosity is an averaged viscosity of the cytoplasmic portion, neglecting the complex internal structures such as the nucleus.

Neural network-based pattern recognitions were conducted based on a ‘Neural Network Pattern Recognition App’ (Matlab 2010, MathWorks, Natick, MA, USA) to differentiate the cytoplasmic viscosity of (1) H1299 cells and A549 cells, (2) H1299 cells with and without compromised cytoskeletons, and (3) H1299 cells under the aspiration pressure of 10 or 5 kPa.

The app employs a two-layer (hidden and output layer) feed-forward neural network, with sigmoid hidden and softmax output neurons. In this study, for the classification of two cell types, the values of cytoplasmic viscosity were used as inputting datasets and the corresponding 0/1 matrix (0 represents cell type one and 1 represents cell type 2) was used as the output matrix. For each classification, the complete dataset was divided into training data (70%, percentage of dataset presented to the network for its adjustment based on generated errors in the training step), validation data (15%, percentage of dataset used to measure network generalization, which can be halted if no further improvements can be made) and testing data (15%, percentage of dataset used to independently measure the network performance after training). As to the results, the neural network-based pattern recognition generated an equation capable of translating the values of cytoplasmic viscosity to 0/1 matrix with the corresponding accuracies shown in a confusion matrix with three kinds of data (training, validation and test) combined. More specifically, the red, green and blue squares represented incorrect responses, correct responses and the overall accuracies (successful classification rates in this study) [35].

Note that in neural network-based pattern recognition, a successful classification rate of 50% means that two datasets cannot be classified at all while a successful classification rate of 100% means that two datasets can be differentiated with a 100% confidence. Thus, as the successful classification rate increases from 50 to 100%, more and more significant differences between the two datasets can be located.

In comparison to conventional approaches for data classification, neural network-based pattern recognition was used in this study for the following two reasons. (1) Neural network-based pattern recognition is capable of providing a successful classification rate to differentiate two types of datasets. For an incoming cell without a pre-known cell type, the successful classification rate can function as a confidence level for us to determine its cell type. Although many statistical approaches (e.g. Student's *t*-test) can evaluate the distribution differences between two cell types, they cannot produce successful classification rates, and thus they cannot be used for further differentiations of incoming new cells. (2) Neural network-based pattern recognition can classify samples without strict distribution requirements such as normal distributions required by the Student's *t*-test.

## 3. Results and discussion

Cytoplasmic viscosity is a key intrinsic cellular mechanical parameter describing the cytoskeleton status of single cells [34], which can be mainly determined by (i) viscosity sensitive fluorescent probes as a biochemical approach, and (ii) micropipette aspiration as a biophysical approach. For the biochemical approach, probes with fluorescent intensities regulated by the viscosity of surrounding medium are injected within biological cells, and the corresponding images are obtained by fluorescent microscopy to determine viscosity distributions within single cells [36,37]. However, due to the lack of calibration approaches (i.e. cells with pre-determined values of cytoplasm viscosity), the biochemical approach cannot effectively correlate raw fluorescent intensities with the actual values of cytoplasm viscosity. On the other hand, micropipette aspiration has been used to characterize single-cell cytoplasmic viscosity from the perspective of biophysics [38]. Based on this approach, actual values of cytoplasm viscosity from various types of blood cells were obtained [29–31,39–41] while the corresponding values of tumour cells were unknown. Thus, in this study, the cytoplasmic viscosity of single tumour cells was characterized and compared to investigate the possibility of using it in tumour cell type classification and status evaluation.

**Figure 2.**
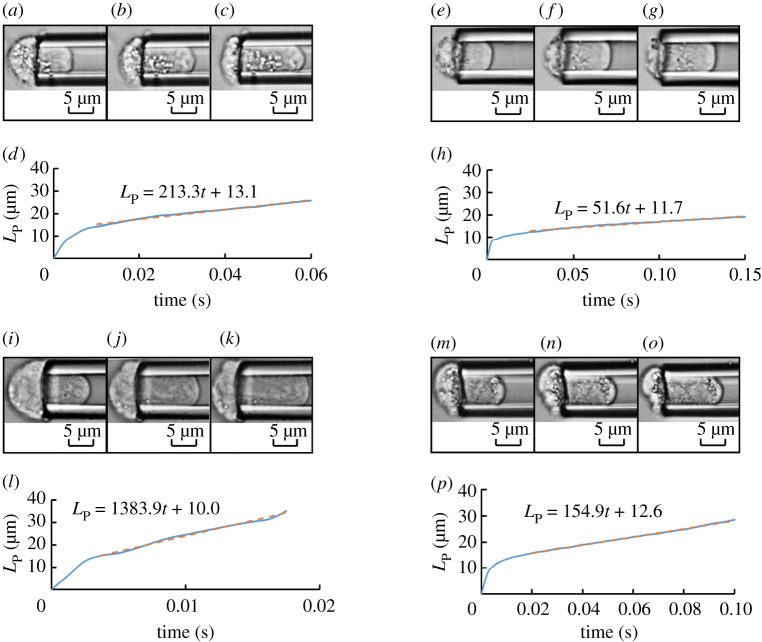
Raw experimental data include microscopic pictures of cellular entries into glass pipettes with quantified aspiration lengths as function of time where (*a*)–(*d*), (*e*)–(*h*), (*i*)–(*l*) and (*m*)–(*p*) show representative cells of H1299 at −10 kPa, A549 at −10 kPa, CD-treated H1299 at −10 kPa and H1299 at −5 kPa, respectively.

Figure 2 shows raw experimental data including microscopic pictures of cellular entries into glass pipettes with quantified aspiration lengths as function of time where Figure 2(*a*)–(*d*), (*e*)–(*h*), (*i*)–(*l*) and (*m*)–(*p*) show representative cells of H1299 at −10 kPa, A549 at −10 kPa, CD-treated H1299 at −10 kPa and H1299 at −5 kPa, respectively.

By interpreting the curves of the aspiration length as a function of time, the cellular entry processes can be divided into two sections. In section I, *L*_p_(*t*) was shown to increase rapidly in response to aspiration, which can be attributed to the elastic response of cells. This behaviour was investigated in many previous studies [22,42] and is not the focus of this work. In section II, a linear increase in *L*_p_(*t*) as a function of time was noted, suggesting the viscous properties of cytoplasm. By conducting a linear curve fitting for section II of the *L*_P_ versus time curve, the aspiration rates, d*L*_p_(*t*)/d*t*, were obtained.

**Figure 3.**
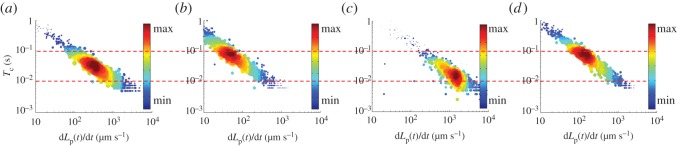
Scatter plots of aspiration time (*T*_c_, the time duration from the instant when the cell first contacted the micropipette to the instant when the cell fully entered the micropipette) versus aspiration rate (d*L*_p_(*t*)/d*t*) for H1299 cells at −10 kPa (*n*_cell_ = 652, (*a*)), A549 cells at −10 kPa (*n*_cell_ = 785, (*b*)), CD-treated H1299 cells at −10 kPa (*n*_cell_ = 651, (*c*)) and H1299 cells at −5 kPa (*n*_cell_ = 600, (*d*)).

Figure 3 shows the scatter plots of the aspiration time (*T*_c_, the time duration from the instant when the cell first contacted the micropipette to the instant when the cell fully entered the micropipette) versus aspiration rate (d*L*_p_(*t*)/d*t*) for H1299 cells at −10 kPa (*n*_cell_ = 652, figure 3*a*), A549 cells at −10 kPa (*n*_cell_ = 785, figure 3*b*), CD-treated H1299 cells at −10 kPa (*n*_cell_ = 651, figure 3*c*) and H1299 cells at −5 kPa (*n*_cell_ = 600, figure 3*d*). More specifically, *T*_c_ was quantified as 0.06 ± 0.11 s for H1299 cells at −10 kPa, 0.19 ± 0.33 s for A549 cells at −10 kPa, 0.04 ± 0.10 s for CD-treated H1299 cells at −10 kPa and 0.15 ± 0.24 s for H1299 cells at −5 kPa. When the time gaps among incoming cells were taken into consideration, the approach can produce a throughput in the range of 1 cell s^−1^. Further optimization of cell suspension densities may potentially increase the throughput to the order of 10 cells s^−1^.

As intermediate parameters, *T*_c_ and d*L*_p_(*t*)/d*t* depend on cell sizes, mechanical properties and aspiration pressures. When untreated H1299 cells possessing similar cell sizes and mechanical properties but under different aspiration pressure (−10 versus −5 kPa) were compared, the H1299 cells under a higher aspiration pressure (−10 kPa) exhibit lower *T*_c_ and higher d*L*_p_(*t*)/d*t* than the H1299 cells under −5 kPa. In addition, when untreated H1299 cells were compared with CD-treated H1299 cells, both with similar cell sizes and under identical aspiration pressures (−10 kPa), higher *T*_c_ and lower d*L*_p_(*t*)/d*t* of the untreated H1299 cells were observed in comparison to CD-treated H1299 cells with compromised cytoskeletons and decreased mechanical properties.

**Figure 4.**
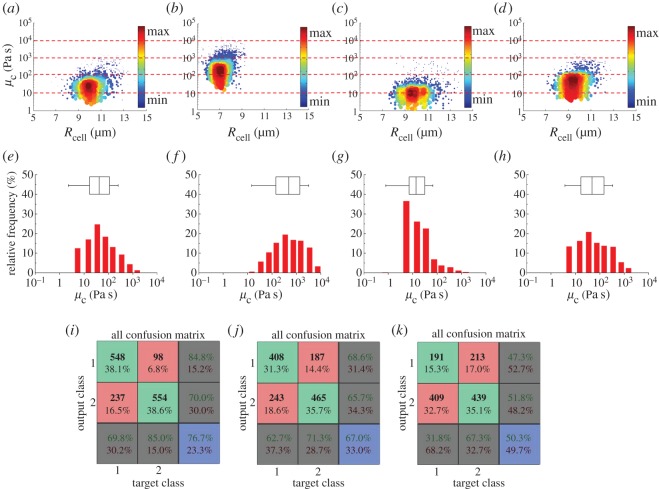
Scatter plots of *μ*_c_ versus *R*_cell_ of H1299 cells at −10 kPa (*n*_cell_ = 652) (*a*), A549 cells at −10 kPa (*n*_cell_ = 785) (*b*), CD-treated H1299 cells at −10 kPa (*n*_cell_ = 651) (*c*) and H1299 cells at −5 kPa (*n*_cell_ = 600) (*d*). Distribution percentages of *μ*_c_, with different peaks located in 10–50 Pa s for H1299 cells at −10 kPa (*e*), 200–500 Pa s for A549 cells at −10 kPa (*f*), 1–10 Pa s for CD-treated H1299 cells at −10 kPa (*g*) and 20–50 Pa s for H1299 cells at −5 kPa (*h*). Confusion matrix of neural network produced successful classification rates of 76.7% for H1299 versus A549 (*i*), 67.0% for H1299 versus drug-treated H1299 (*j*) and 50.3% for H1299 at −5 and −10 kPa (*k*).

As shown in figure 2, a linear increase of *L*_p_(*t*) as a function of time was observed, indicating that the cellular aspiration process can be effectively captured by a viscous droplet model. Therefore, the theoretical Newtonian fluid model was used to model the cell during the aspiration process, which translated the aspiration rate d*L*_p_(*t*)/d*t* into the cytoplasmic viscosity *μ*_c_. Figure 4*a–d* shows the scatter plots of *μ*_c_ versus *R*_cell_ of H1299 cells at −10 kPa (*n*_cell_ = 652, figure 4*a*), A549 cells at −10 kPa (*n*_cell_ = 785, figure 4*b*), CD-treated H1299 cells at −10 kPa (*n*_cell_ = 651, figure 4*c*) and H1299 cells at −5 kPa (*n*_cell_ = 600, figure 4*d*, respectively. Figure 4*e*–*h* shows distribution percentages of *μ*_c_, with different peaks for the four sets of data, i.e. 10–50 Pa s for H1299 cells at −10 kPa, 200–500 Pa s for A549 cells at −10 kPa, 1–10 Pa s for CD-treated H1299 cells at −10 kPa and 20–50 Pa s for H1299 cells at −5 kPa, respectively. Furthermore, three quartiles and the quartile coefficient of dispersion were quantified as 16.7 Pa s, 42.1 Pa s, 110.3 Pa s and 74% for H1299 cells at −10 kPa; 144.8 Pa s, 489.8 Pa s, 1390.7 Pa s, and 81% for A549 cells at −10 kPa; 7.1 Pa s, 13.7 Pa s, 31.5 Pa s, and 63% for CD-treated H1299 cells at −10 kPa; and 16.9 Pa s, 48.2 Pa s, 150.2 Pa s, and 80% for H1299 cells at −5 kPa. These results reveal the significant variations of cytoplasmic viscosity within the same cell types.

As to comparisons between different cell types, significant differences in cytoplasmic viscosity were noted. More specifically, values of cytoplasmic viscosity for H1299 versus A549 cells were 16.7 versus 144.8 Pa s for the first quartile, 42.1 versus 489.8 Pa s for the median of the data and 110.3 versus 1390.7 Pa s for the third quartile, respectively. Furthermore, neural network-based pattern recognition produced successful classification rates of 76.7% for H1299 versus A549 (figure 4*i*), further indicating the potential role of cytoplasmic viscosity in cell type classification. Note that since the values of cytoplasmic viscosity obtained in this study cannot meet normal distributions, Student's *t*-tests and other statistical approaches were not used for the cell type classification.

As to the comparisons of H1299 cells with and without the treatment of CD, significant decreases in cytoplasmic viscosity were noted for the H1299 cells following the treatment of CD. More specifically, cytoplasmic viscosity of H1299 cells with and without the treatment of CD was quantified as 7.1 versus 16.7 Pa s for the first quartile, 13.7 versus 42.1 Pa s for the median of the data and 31.5 versus 110.3 Pa s for the third quartile, respectively. Furthermore, neural network-based pattern recognition produced successful classification rates of 67.0% for H1299 cells with and without the CD treatment (figure 4*j*), further confirming cytoskeleton compromises due to the CD treatment.

As to the comparison of H1299 cells under −10 or −5 kPa, a successful classification rate of 50.3% was collected (figure 4*k*). In addition, comparable values of cytoplasmic viscosity for H1299 cells under −10 and −5 kPa were noted, which were 16.7 versus 16.9 Pa s for the first quartile, 42.1 versus 48.2 Pa s for the median of the data and 110.3 versus 150.2 Pa s for the third quartile, respectively. These results suggested that based on the current approach of micropipette aspiration, the characterization of cytoplasmic viscosity was independent of the aspiration pressures and thus the quantified values of cytoplasmic viscosity were trustworthy.

## 4. Conclusion

In this study, the cytoplasmic viscosity of tumour cells was characterized by micropipette aspiration. High quartile coefficients of dispersion (approx. 70%) were observed, indicating significant variations of cytoplasmic viscosity within the same cell type. Successful classification rates based on neural network (approx. 70%) were quantified between H1299 and A549 cells, H1299 cells with and without the treatment of CD, suggesting the potential role of cytoplasmic viscosity in cell type classification and cell status evaluation. Future developments may focus on the applications of micropipette aspirations to characterize tumour cells from patients continuously. In order to meet this demand, technical developments in processing throughput and stability of micropipette aspirations have to be made.

## Data accessibility

Data are available from Dryad Digital Repository: http://dx.doi.org/10.5061/dryad.46kk44r [43].

## References

[1] Ethier CR, Simmons CA 2007 Introductory biomechanics: from cells to organisms (Cambridge texts in biomedical engineering). Cambridge, UK: Cambridge University Press.

[2] Fletcher DA, Mullins RD 2010 Cell mechanics and the cytoskeleton. Nature 463, 485–492. (10.1038/nature08908) 20110992 PMC2851742

[3] Di Carlo D 2012 A mechanical biomarker of cell state in medicine. J. Lab. Automat. 17, 32–42. (10.1177/2211068211431630) 22357606

[4] Lee GYH, Lim CT 2007 Biomechanics approaches to studying human diseases. Trends Biotechnol. 25, 111–118. (10.1016/j.tibtech.2007.01.005) 17257698

[5] Chen J et al. 2016 Single-cell mechanical properties: label-free biomarkers for cell status evaluation. In Essentials of single-cell analysis: concepts, applications and future prospects (eds Tseng F-G, Santra TS), pp. 213–234. Berlin, Germany: Springer.

[6] Van Vliet KJ, Bao G, Suresh S 2003 The biomechanics toolbox: experimental approaches for living cells and biomolecules. Acta Mater. 51, 5881–5905. (10.1016/j.actamat.2003.09.001)

[7] Lim CT, Zhou EH, Li A, Vedula SRK, Fu HX 2006 Experimental techniques for single cell and single molecule biomechanics. Mater. Sci. Eng. C 26, 1278–1288. (10.1016/j.msec.2005.08.022)

[8] Addae-Mensah KA, Wikswo JP 2008 Measurement techniques for cellular biomechanics *in vitro*. Exp. Biol. Med. 233, 792–809. (10.3181/0710-MR-278) PMC4156015 18445766

[9] Lehenkari PP, Charras GT, Nykanen A, Horton MA 2000 Adapting atomic force microscopy for cell biology. Ultramicroscopy 82, 289–295. (10.1016/S0304-3991(99)00138-2) 10741681

[10] Charras GT, Horton MA 2002 Single cell mechanotransduction and its modulation analyzed by atomic force microscope indentation. Biophys. J. 82, 2970–2981. (10.1016/S0006-3495(02)75638-5) 12023220 PMC1302085

[11] Radmacher M 2002 Measuring the elastic properties of living cells by the atomic force microscope. Methods Cell Biol. 68, 67–90. (10.1016/S0091-679X(02)68005-7) 12053741

[12] Alonso JL, Goldmann WH 2003 Feeling the forces: atomic force microscopy in cell biology. Life Sci. 72, 2553–2560. (10.1016/S0024-3205(03)00165-6) 12672501

[13] Costa KD 2003 Single-cell elastography: probing for disease with the atomic force microscope. Dis. Markers 19, 139–154. (10.1155/2004/482680) 15096710 PMC3850842

[14] Costa KD 2006 Imaging and probing cell mechanical properties with the atomic force microscope. Methods Mol. Biol. 319, 331–361. (10.1007/978-1-59259-993-6_17) 16719364

[15] Kuznetsova TG, Starodubtseva MN, Yegorenkov NI, Chizhik SA, Zhdanov RI 2007 Atomic force microscopy probing of cell elasticity. Micron 38, 824–833. (10.1016/j.micron.2007.06.011) 17709250

[16] Lekka M, Laidler P 2009 Applicability of AFM in cancer detection. Nat. Nanotechnol. 4, 72 (10.1038/nnano.2009.004) 19197298

[17] Kirmizis D, Logothetidis S 2010 Atomic force microscopy probing in the measurement of cell mechanics. Int. J. Nanomed. 5, 137–145. (10.2147/IJN.S5787) PMC2865008 20463929

[18] Guo Q, Xia Y, Sandig M, Yang J 2012 Characterization of cell elasticity correlated with cell morphology by atomic force microscope. J. Biomech. 45, 304–309. (10.1016/j.jbiomech.2011.10.031) 22115064

[19] Shi X, Zhang X, Xia T, Fang X 2012 Living cell study at the single-molecule and single-cell levels by atomic force microscopy. Nanomedicine 7, 1625–1637. (10.2217/nnm.12.130) 23148543

[20] Zheng Y, Nguyen J, Wei Y, Sun Y 2013 Recent advances in microfluidic techniques for single-cell biophysical characterization. Lab. Chip 13, 2464–2483. (10.1039/c3lc50355k) 23681312

[21] Cross SE, Jin YS, Rao J, Gimzewski JK 2007 Nanomechanical analysis of cells from cancer patients. Nat. Nanotechnol. 2, 780–783. (10.1038/nnano.2007.388) 18654431

[22] Hochmuth RM 2000 Micropipette aspiration of living cells. J. Biomech. 33, 15–22. (10.1016/S0021-9290(99)00175-X) 10609514

[23] Chen Y, Liu B, Xu G, Shao J-Y 2009 Validation, in-depth analysis, and modification of the micropipette aspiration technique. Cell. Mol. Bioeng. 2, 351–365. (10.1007/s12195-009-0071-9) 20333318 PMC2843006

[24] Ward KA, Li WI, Zimmer S, Davis T 1991 Viscoelastic properties of transformed cells: role in tumor cell progression and metastasis formation. Biorheology 28, 301–313. (10.3233/BIR-1991-283-419) 1932719

[25] Thoumine O, Ott A 1997 Comparison of the mechanical properties of normal and transformed fibroblasts. Biorheology 34, 309–326. (10.3233/BIR-1997-344-505) 9578806

[26] Wu ZZ, Zhang G, Long M, Wang HB, Song GB, Cai SX 2000 Comparison of the viscoelastic properties of normal hepatocytes and hepatocellular carcinoma cells under cytoskeletal perturbation. Biorheology 37, 279–290. 11145074

[27] Zhang G, Long M, Wu ZZ, Yu WQ 2002 Mechanical properties of hepatocellular carcinoma cells. World J. Gastroenterol. 8, 243–246. (10.3748/wjg.v8.i2.243) 11925600 PMC4658359

[28] Anderson K, Li W-I, Cezeaux J, Zimmer S 1991 *In vitro* studies of deformation and adhesion properties of transformed cells. Cell Biophys. 18, 81–97. (10.1007/BF02989808) 1726528

[29] Needham D, Hochmuth RM 1990 Rapid flow of passive neutrophils into a 4 microns pipet and measurement of cytoplasmic viscosity. J. Biomech. Eng. 112, 269–276. (10.1115/1.2891184) 2214708

[30] Tsai MA, Frank RS, Waugh RE 1993 Passive mechanical behavior of human neutrophils: power-law fluid. Biophys. J. 65, 2078–2088. (10.1016/S0006-3495(93)81238-4) 8298037 PMC1225943

[31] Drury JL, Dembo M 2001 Aspiration of human neutrophils: effects of shear thinning and cortical dissipation. Biophys. J. 81, 3166–3177. (10.1016/S0006-3495(01)75953-X) 11720983 PMC1301777

[32] Wakatsuki T, Schwab B, Thompson NC, Elson EL 2001 Effects of cytochalasin D and latrunculin B on mechanical properties of cells. J. Cell Sci. 114, 1025–1036. 11181185 10.1242/jcs.114.5.1025

[33] Gonzalez RC, Woods RE 2007 Digital image processing, 3rd edn pp. 1160–1165. Englewood Cliffs, NJ: Prentice-Hall, Inc.

[34] Lim CT, Zhou EH, Quek ST 2006 Mechanical models for living cells: a review. J. Biomech. 39, 195–216. (10.1016/j.jbiomech.2004.12.008) 16321622

[35] Xu H, Lai JG, Liu JY, Cao N, Zhao J 2013 Neural network pattern recognition and its application. Adv. Mater. Res. 756–759, 2438–2442. (10.4028/www.scientific.net/AMR.756-759.2438)

[36] Lee SC et al. 2018 Fluorescent molecular rotors for viscosity sensors. Chemistry. 24, 13 706–13 718. (10.1002/chem.201801389) 29700889

[37] Kuimova MK 2012 Mapping viscosity in cells using molecular rotors. Phys. Chem. Chem. Phys. 14, 12 671–12 686. (10.1039/c2cp41674c) 22806312

[38] Lee LM, Liu AP 2014 The application of micropipette aspiration in molecular mechanics of single cells. J. Nanotechnol. Eng. Med. 5, 0 408 011–0 408 016. 10.1115/1.4029936 PMC4476029 26155329

[39] Evans E, Yeung A 1989 Apparent viscosity and cortical tension of blood granulocytes determined by micropipet aspiration. Biophys. J. 56, 151–160. (10.1016/S0006-3495(89)82660-8) 2752085 PMC1280460

[40] Tran-Son-Tay R, Needham D, Yeung A, Hochmuth RM 1991 Time-dependent recovery of passive neutrophils after large deformation. Biophys. J. 60, 856–866. (10.1016/S0006-3495(91)82119-1) 1742456 PMC1260136

[41] Hochmuth RM, Ting-Beall HP, Beaty BB, Needham D, Tran-Son-Tay R 1993 Viscosity of passive human neutrophils undergoing small deformations. Biophys. J. 64, 1596–1601. (10.1016/S0006-3495(93)81530-3) 8324194 PMC1262487

[42] Luo YN et al. 2014 A constriction channel based microfluidic system enabling continuous characterization of cellular instantaneous Young's modulus. Sens. Actuat. B 202, 1183–1189. (10.1016/j.snb.2014.05.028)

[43] Wang K et al. 2019 Data from: Characterization of cytoplasmic viscosity of hundreds of single tumour cells based on micropipette aspiration Dryad Digital Repository. (10.5061/dryad.46kk44r) PMC6458365 31032026

